# A case of Crohn's disease in a patient with megacystis microcolon intestinal hypoperistalsis syndrome

**DOI:** 10.1002/jpr3.70015

**Published:** 2025-03-10

**Authors:** Caroline Chinchilla Putzeys, Trenton House, Janet Iurilli, Ana Gomez, Shipra Garg, Elisa Wershba, Gary Silber, Mitchell Shub, Dana Williams

**Affiliations:** ^1^ Department of Gastroenterology Phoenix Children's Hospital Phoenix Arizona USA; ^2^ University of Arizona College of Medicine – Phoenix Phoenix Arizona USA; ^3^ Department of Pathology Phoenix Children's Hospital Phoenix Arizona USA; ^4^ Department of Rheumatology Phoenix Children's Hospital Phoenix Arizona USA

**Keywords:** inflammatory bowel disease, MMHS, motility

## Abstract

Megacystis microcolon intestinal hypoperistalsis syndrome (MMIHS) is a rare congenital condition resulting in symptoms of bowel and bladder pseudo‐obstruction. It carries severe morbidity and mortality; a minority of patients survive to adulthood. Recurrent bowel surgeries, small intestinal bacterial overgrowth, and slowed intestinal transit in MMIHS could serve as potential risk factors for chronic inflammation of the intestinal mucosa, which has been associated with risk of inflammatory bowel disease. In this case report, we detail the unusual presentation of a patient diagnosed with both MMIHS and inflammatory bowel disease.

## 1 INTRODUCTION

Megacystis microcolon intestinal hypoperistalsis syndrome (MMIHS) is a rare congenital myopathic condition affecting the smooth muscle of gastrointestinal and urinary tracts characterized by symptoms of bowel and bladder obstruction without mechanical obstruction.^1^, ^2^ The condition has a female preponderance, with fewer than 800 cases reported since 1976.^1^ Contemporary studies report 5‐year survival rates of 63%, decreasing to 57% at 10 years.^3^ Complications include chronic intestinal pseudo‐obstruction (CIPO), intestinal diversion, ileostomy/colostomy, and gastrostomy/jejunostomy for nutrition and decompression.^4^ Most patients require parenteral nutrition (PN), predisposing them to central line infections, cholestasis, and hepatic failure.^1^, ^2^, ^3^, ^4^ Consequently, MMIHS is associated with high early mortality and complex management, few patients reach adolescence or early adulthood.

We present a case of a patient with MMIHS who was diagnosed with Crohn's Disease in his teenage years.

## 2 CASE REPORT

A full‐term Hispanic male was referred to our institution in early infancy with chronic bowel and bladder pseudo‐obstruction. He failed to pass meconium within 24 h of birth, developing abdominal distension, constipation, and malnutrition. At 10 months old antroduodenal and colonic manometry revealed myopathy, and radiological evaluation confirmed MMIHS. A gastrostomy tube was placed for nutritional support and decompression.

Managed conservatively until the age of 12 years, the patient underwent repeated surgeries for hydroureteronephrosis and central catheter placement for partial PN following subtotal colectomy and jejunostomy. By age 15, multiple recurrent small bowel volvuli requiring surgical detorsion culminated in a small bowel resection, ileostomy creation, and PN dependence.

**Figure 1:**
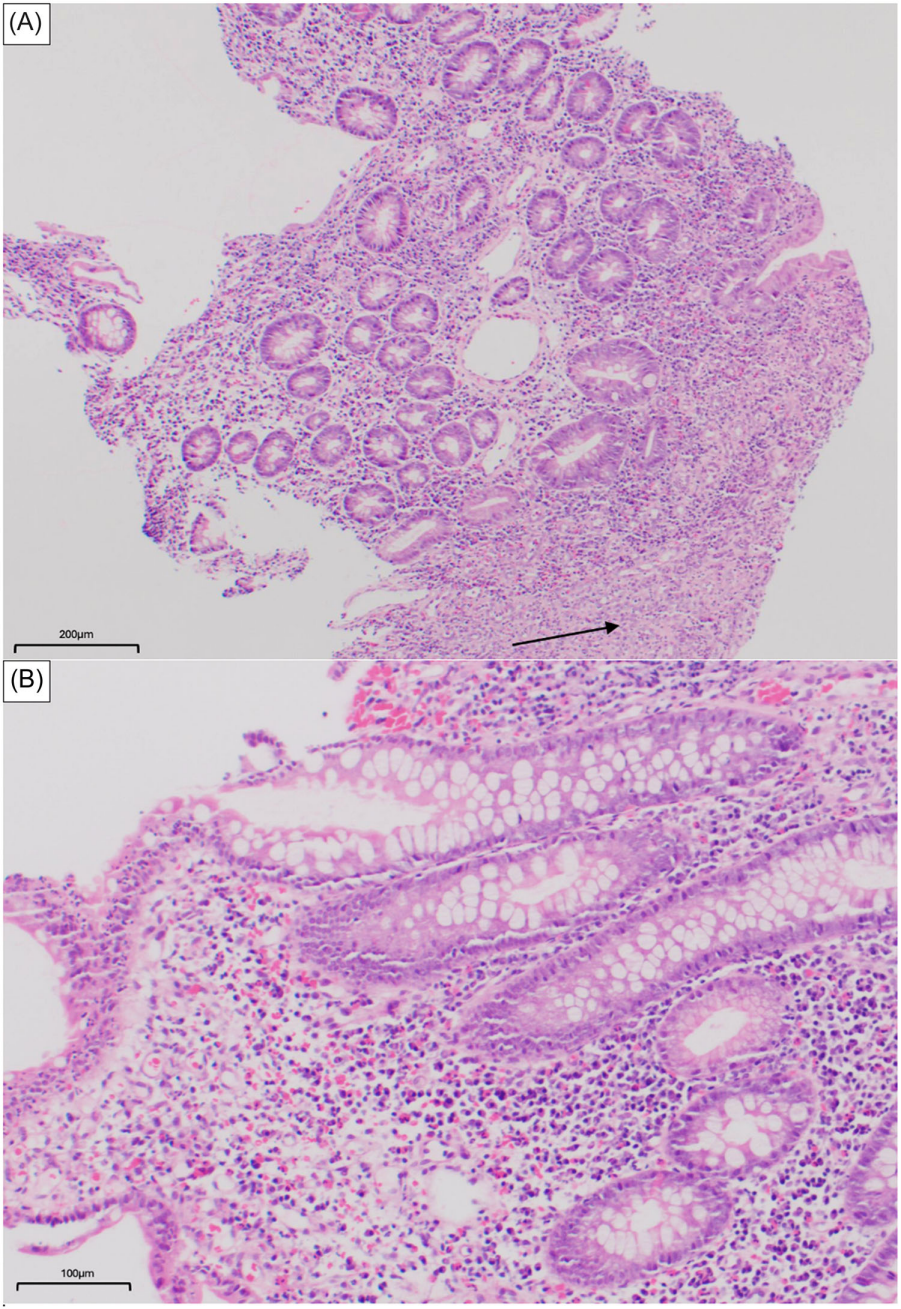
(A) Terminal ileum 10X (H&E). Small bowel mucosa with villous and crypt architectural disarray, expansion of the lamina propria by mixed inflammatory infiltrate. Ulcer bed showing inflamed granulation tissue (arrow). (B) Terminal ileum 20X (H&E). Small bowel mucosa with crypt architectural disarray and expansion of the lamina propria by mixed inflammatory infiltrate. H&E, hematoxylin and eosin.

At 16 years, the patient experienced high ileostomy output unrelated to infection and refractory to small intestinal bacterial overgrowth (SIBO) treatment. He also developed debilitating back pain, limiting ambulation. Rheumatology evaluations with MRI revealed sacroiliitis. Testing confirmed HLAB27+ spondylarthritis, raising suspicion for concomitant inflammatory bowel disease (IBD). Endoscopic biopsies revealed active ileitis (Figure 1), Paris classification A1b L4a B1 G0 suggestive of Crohn's disease (CD). IBD serology was positive for anti‐OmpC IgA, IBD‐specific pANCA, and ASCA IgA; and genetic mutations in ATG16L1, ECM1, and NKX2‐3 linked to CD were identified. Magnetic resonance enterography (MRE) did not reveal strictures or fistulae. Treatment was initiated with adalimumab and intermittent corticosteroids to for ankylosing spondylitis and CD.

At 17 years, the patient began receiving intrapyloric botulinum toxin injections every 4 months for gastroparesis. Over the next year, he required two bilateral hip joint aspirations. Due to repeated exacerbations of ankylosing spondylitis, rheumatology, in consultation with gastroenterology, switched his biologic to certolizumab pegol for better sacroiliitis control.

Between 12 and 19 years, the patient's height z score declined from −0.92 to −2.15 (indicating short stature). Between 19 and 20, the patient exhibited severe malnutrition with inflammation, losing 13.5% of his usual body weight over 12 months despite receiving PN. Physical findings included significant muscle and subcutaneous fat loss.

At 19, endoscopic biopsies revealed acute and chronic ileitis, his first flare of CD since diagnosis. Fecal calprotectin measured >800 µg/g (normal <50 µg/g). Treatment was augmented with methotrexate, normalizing fecal calprotectin and improving enteral symptoms. In the subsequent 2 years the patient had two hospitalizations for pancreatitis and required two ostomy dilations for small bowel strictures.

At 22, two additional CD flares necessitated a switch to ustekinumab. At 23, he was switched to ixekizumab as CD was quiescent, and his major issue was arthritic flares. He was monitored closely with ileoscopies, showing no evidence of inflammation. At 24, he was transitioned to adult medicine and has since been switched between multiple biologics to target flares of either spondyloarthritis or CD.

**Figure 2:**
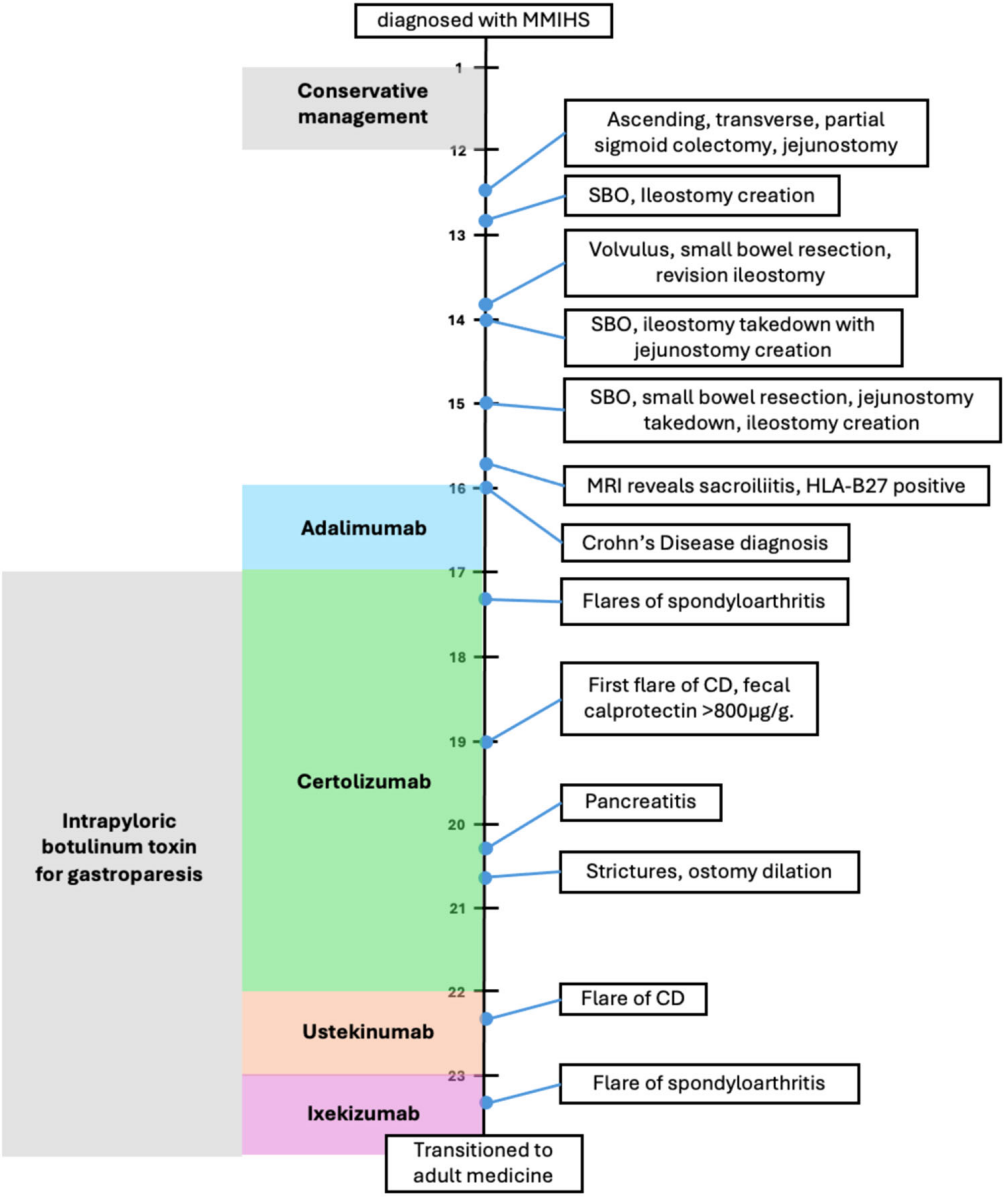
Timeline of patient case. CD, Crohn's disease; MMIHS, Megacystis microcolon intestinal hypoperistalsis syndrome; MRI, magnetic resonance imgaing; SBO, small bowel obstruction.

Selected parts of the patient's history are presented chronologically in Figure 2.

## 3 DISCUSSION

MMIHS is a rare genetic disease with a poorly understood pathophysiology is poorly understood, with varied histologic findings of ganglion cells and neuropeptides in the bowel smooth muscle reported.^5^ Improved PN and surgical management have improved survival, allowing more patients are reaching adulthood and revealing associated conditions including mucosal disease, previously unrecognized.^3^

This is an unusual case of IBD in MMIHS. IBD pathogenesis invokes genetic predisposition, loss of intestinal barrier with aberrant immune response, gut microbiome alterations, and environmental factors. This patient has multiple risk factors predisposing him to IBD. HLA‐B27 positivity is associated with IBD and spondyloarthritis.^6^ Our patient presented with extra‐gastrointestinal symptoms (i.e., rheumatologic disease) related to HLA B27 positivity. Recurrent bowel surgeries, short gut syndrome, SIBO, and affected intestinal transit in MMIHS are potential risk factors for chronic mucosal inflammation.^7^ Recent literature reports both short bowel syndrome and IBD share underlying intestinal mucosal immune dysfunction and altered gut microbiota.^8^ Contemporary studies cataloging genetic mutations in MMIHS and IBD report an association with MYL9 mutations on chromosome 20q.^9^ No studies have investigated shared genetic alterations between the two conditions.

Several studies in patients on chronic PN and frequent antibiotic use document that gut microbiota changes are associated with IBD development.^9^, ^10^ We speculate that a combination of these factors occurred in our patient. An altered gut microbiome, leading to increased intestinal permeability, and triggering dysregulated immune responses in a genetically predisposed patient is a potential mechanism of IBD development.^11^, ^12^ Chronic mucosal inflammation screening and endoscopic assessment for IBD should be considered in all patients with MMIHS with worsening gastrointestinal symptoms refractory to medical management with or without rheumatologic concerns. Chronic gastrointestinal symptomatology in these children could delay a diagnosis of IBD without a high index of suspicion.

A thorough review of the literature did not reveal any documented cases of IBD as a long‐term complication of MMIHS. Our patient is one of a minority of reported MMIHS patients surviving to adulthood and is the first with a concomitant CD diagnosis and HLAB27+ spondylarthritis.

## CONFLICT OF INTEREST STATEMENT

The authors declare no conflict of interest.

## ETHICS STATEMENT

Phoenix Children's Hospital Institutional Review Board approved this project. Approval number IRB‐24‐070. Written informed consent was received from the patient.
